# Circular RNA Foxo3 enhances progression of ovarian carcinoma cells

**DOI:** 10.18632/aging.203550

**Published:** 2021-09-23

**Authors:** Li Wang, Jing Chen, Chunhua Lu

**Affiliations:** 1 Department of Gynaecology, The First Affiliated Hospital of Jinzhou Medical University, Jinzhou 121000, China; 2 Department of Urinary Surgery, The First Affiliated Hospital of Jinzhou Medical University, Jinzhou 121000, China

**Keywords:** ovarian carcinoma, progression, Foxo3, miR-422a, PLP2, exosome

## Abstract

Background: Ovarian carcinoma (OC) is the deadliest gynecologic malignancy in females worldwide. Circular RNA Foxo3 (Foxo3) plays essential roles in various cancers. However, the detailed function of Foxo3 in OC remains unclear. This study aimed to investigate the role of Foxo3 in OC and the underlying molecular mechanism.

Methods: The abundance of Foxo3 was detected in OC cell lines by qPCR. Lentivirus transduction, CCK-8, wound healing assays, transwell migration and invasion assays, luciferase reporter assay, western blotting, fluorescence *in situ* hybridization (FISH), transmission electron microscopy, nanoparticle tracking analysis, and bioinformatics analysis were performed to investigate the underlying mechanism.

Results: The results demonstrated that Foxo3 was significantly upregulated in OC cell lines. Overexpression and knockdown of Foxo3 promoted and inhibited the proliferation, migration, and invasion of OC cells, respectively. Foxo3 could bind to miR-422a to negatively regulate miR-422a expression. Also, proteolipid protein 2 (PLP2) was a targeting gene of miR-422a. Additionally, Foxo3 was highly expressed in exosomes derived from OC cells. Furthermore, Foxo3 could be shuttled to OC cells by exosomes and promoted OC progression.

Conclusions: Foxo3 promoted OC progression through exosome-mediated intercellular interaction to target miR-422a/PLP2 axis. Foxo3 may serve as a potential biomarker for OC.

## INTRODUCTION

Ovarian carcinoma (OC) is the most devastating gynecologic malignancy among females in the world [1]. It is estimated that there were 295,414 new OC cases and 184,799 OC-associated death in 2018 worldwide [2]. From 2000 to 2003, in China, OC emerged as the first deadly cancer, with around 21.6% mortality among women and the second most wide-spreading cancer following cervical cancer [3]. OC is notably characterized by its high heterogeneity, which complicates early diagnosis and primary prevention [4]. Thus, many patients with OC are already in the advanced stage when diagnosed, leading to a low 5-year survival rate, less than 20% [5]. Currently, surgery, along with chemotherapy, is an effective treatment for OC [6]. However, the prognosis and survival rate of patients with OC are still unsatisfactory due to its high recurrence rate [6]. Therefore, understanding the molecular mechanism underlying OC initiation and progression is an urgent need.

Circular RNAs (circRNAs) are a class of novel non-coding RNAs (ncRNAs) that play essential roles in the various biological processes [7]. In general, circRNAs function as microRNA (miRNA) sponges, competing for miRNAs binding and regulating miRNA expression [8]. It has been widely documented that circRNAs are closely related to tumor occurrence and progression, allowing circRNAs to be promising biomarkers or therapeutic targets for cancers [9]. As one of the well-studied circRNAs in cancers, circRNA forkhead box O3 (Foxo3) acts as either an anti-tumor or pro-tumor regulator in various cancer types. Xing et al. reported that the downregulation of Foxo3 promotes cell growth, migration, and invasion in esophageal squamous cell cancer through targeting miR-23a [10]. Wang et al. demonstrated that the overexpression of Foxo3 enhances bladder cancer cell apoptosis through sponging miR-191-5p [11]. Furthermore, the downregulation of Foxo3 promotes cell proliferation, migration, and invasion in non-small cell lung cancer cells by targeting miR-155 [12]. So far, however, the function of Foxo3 has not been thoroughly investigated in OC.

Exosomes are a class of nano-sized extracellular membranous microvesicles, with 30-150 nm in diameter [13]. Exosomes derived from donating cells can be taken up by distant or neighboring cells, thereby impacting the biological processes of recipient cells [14]. Exosomes have been demonstrated to be associated with the initiation and progression of various cancers and play a critical role in cell-to-cell communication by shuttling functional cargos, such as circRNAs, miRNAs, DNAs, and proteins [15, 16]. Growing evidence suggests that functional molecules, including circRNAs, can be wrapped into exosomes and transported to recipient cells, further influencing cancer progression [17]. Nonetheless, it is unclear whether or not Foxo3 can be shuttled by exosomes between OC cells. Collectively, this study thus aimed to investigate the function of Foxo3 in the progression of OC and underlying mechanisms.

## RESULTS

### Upregulation of Foxo3 promotes OC cell proliferation, migration, and invasion

**Figure 1:**
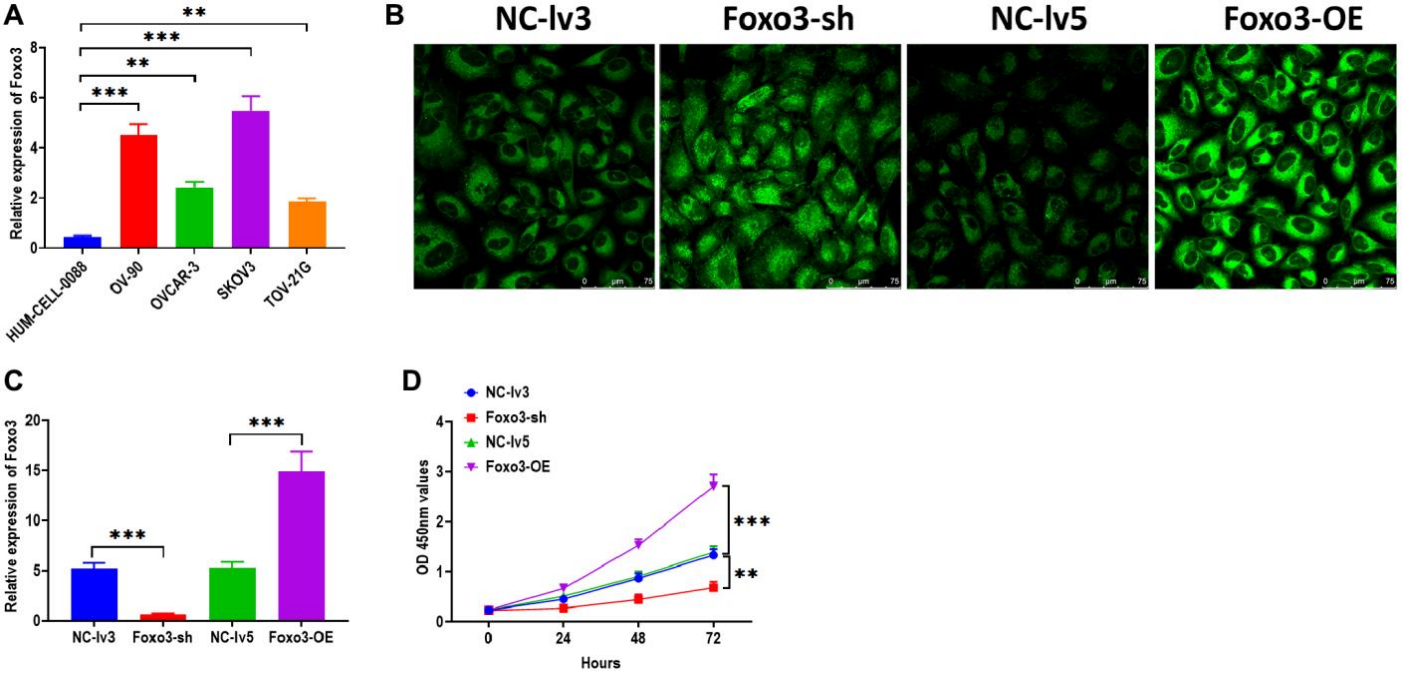
**Upregulation of Foxo3 promotes OC cell proliferation.** (**A**) The expression of Foxo3 in OC and control cells. (**B**) Visualization of lentivirus transfection in SKOV3 cells, as observed by fluorescence microscope. Scale bar = 75 μm. (**C**) Transfection efficiency in Foxo3-knockdown or Foxo3-overexpressing SKOV3 cells. (**D**) Cell proliferation of SKOV3 cells with Foxo3 knockdown or overexpressing, as determined by CCK-8 assay. Data were expressed as means ± standard deviation. For each treatment, three replicates were used. ^**^p < 0.01, ^***^p < 0.001.

**Figure 2:**
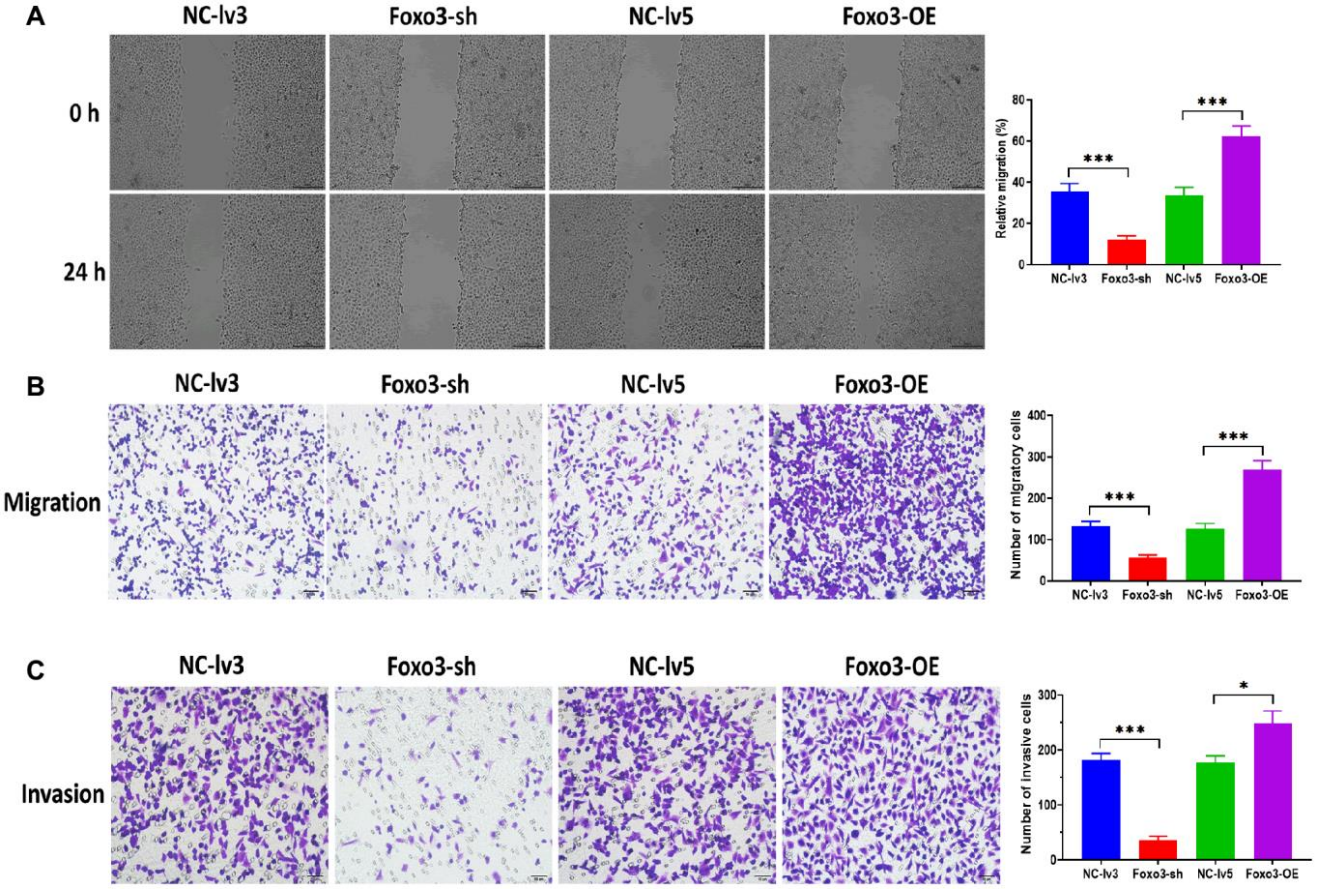
**Upregulation of Foxo3 promotes OC cell migration and invasion.** (**A**) Migration ability of SKOV3 cells with Foxo3 knockdown or overexpressing, as determined by wound healing assay. Scale bar = 200 μm. (**B**) Migration ability of SKOV3 cells with Foxo3 knockdown or overexpressing, as determined by Transwell migration assay. Scale bar = 50 μm. (**C**) Invasion ability of SKOV3 cells with Foxo3 knockdown or overexpressing, as determined by Transwell invasion assay. Scale bar = 50 μm. Data were expressed as means ± standard deviation. For each treatment, three replicates were used. ^*^p < 0.05, ^***^p < 0.001.

To investigate the function of Foxo3 in OC, we first determined the expression of Foxo3 in several OC cell lines SKOV3, TOV-21G, OV-90, and OVCAR-3. Compared with the control cell HUM-CELL-0088, Foxo3 was significantly upregulated in all OC cell lines, of which the most significant upregulation of Foxo3 was observed in SKOV3 cells (Figure 1A). Thus, SKOV3 cells were selected for subsequential experiments. Next, we applied loss/gain-of-function experiments to create SKOV3 cells with Foxo3 knockdown or overexpression. Fluorescence staining assay revealed that the lentiviruses for Foxo3 knockdown or overexpression were successfully transfected into SKOV3 cells (Figure 1B). Meanwhile, the qPCR assay demonstrated that the expression of Foxo3 was notably inhibited in cells transfected with Foxo3-sh vector while increased in cells transfected with Foxo3-OE vector (Figure 1C). Then, we performed a series of functional experiments to investigate the role of Foxo3 in cell proliferation, migration, and invasion. CCK-8 assay showed that SKOV3 cells with Foxo3 knockdown displayed inhibited cell proliferation, whereas the overexpression of Foxo3 significantly increased the proliferation of SKOV3 cells (Figure 1D). Through the wound healing assay, the results revealed that the inhibition of Foxo3 suppressed the migratory ability of SKOV3 cells, and the overexpression of Foxo3 promoted migration (Figure 2A). In transwell migration and invasion assays, SKOV3 cells with Foxo3 knockdown showed decreased migratory and invasive ability, while the opposite results were found in cells with Foxo3 overexpression (Figure 2B and 2C). These results collectively showed that Foxo3 was significantly upregulated in OC cells and might play an essential role in OC progression.

### Foxo3 directly targets miR-422a

**Figure 3:**
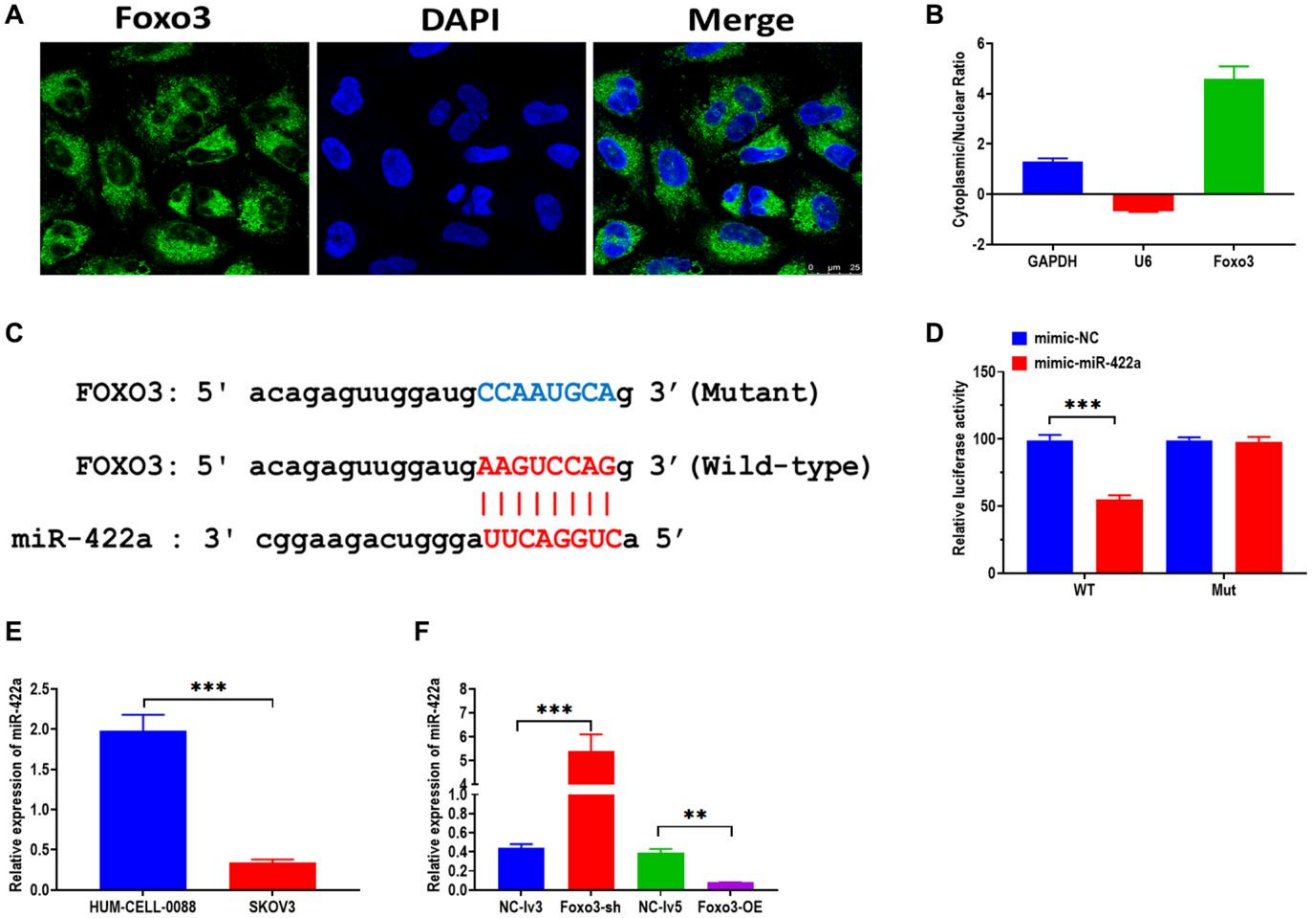
**Foxo3 directly targets miR-422a.** (**A**) Subcellular distribution of Foxo3 in SKOV3 cells, as investigated by fluorescence *in situ* hybridization (FISH) assay. Scale bar = 25 μm. (**B**) Subcellular distribution of Foxo3 in SKOV3 cells, as investigated by subcellular fractionation assay. GAPDH and U6 were used as reference genes for the nuclear and cytoplasmic, respectively. The data were expressed as cytoplasmic/nuclear ratio. (**C**) The putative binding sequence of miR-422a of 3’UTR of Foxo3. The prediction was obtained from StarBase 2.0. (**D**) Luciferase reporter assay in HEK293T cells co-transfected with miR-422a mimics or mimics a negative control plus reporter vector containing wild-type or mutant miR-422a binding sequence 3′-UTR of Foxo3. (**E**) The expression of miR-422a in SKOV3 and control cells. (**F**) The expression of miR-422a in SKOV3 cells with Foxo3 knockdown or overexpressing. Data were expressed as means ± standard deviation. For each treatment, three replicates were used. ^**^p < 0.01, ^***^p < 0.001.

It has been demonstrated that cricRNAs play biological functions by targeting miRNAs in the cytoplasm [18, 19]. To investigate the mechanism underlying the role of Foxo3 in OC, we then determine the subcellular distribution of Foxo3 in SKOV3. By using both FISH and subcellular fractionation assay, the results showed that Foxo3 was primarily distributed in the cytoplasm (Figure 3A and 3B). Subsequently, we performed the bioinformatic analysis in the online tool StarBase 2.0 [20] to predict the potential targeting miRNAs of Foxo3. Among many candidates, Foxo3 had a binding site of miR-422a in the 3′UTR sequence (Figure 3C). As a well-studied regulator, miR-422a was reported to be associated with several cancers [21–23]. Thus, we carried out the luciferase reported assay to verify this prediction. The results showed that the relative luciferase activity was decreased in HEK293T cells co-transfected with miR-422a mimics and vectors containing the wild-type binding site (Figure 3D). Also, luciferase activity did not change in cells co-transfected with miR-422a mimics and vectors containing the mutant binding site. In addition, we found that the level of miR-422a was significantly decreased in SKOV3 relative to HUM-CELL-0088 cells (Figure 3E). Furthermore, the expression of miR-422a was increased in SKOV3 cells with Foxo3 knockdown while decreased in cells with Foxo3 overexpression (Figure 3F). Taken together, these results demonstrate that Foxo3 functions as a miRNA sponge to target miR-422a in OC cells directly.

### miR-422a directly targets PLP2

**Figure 4:**
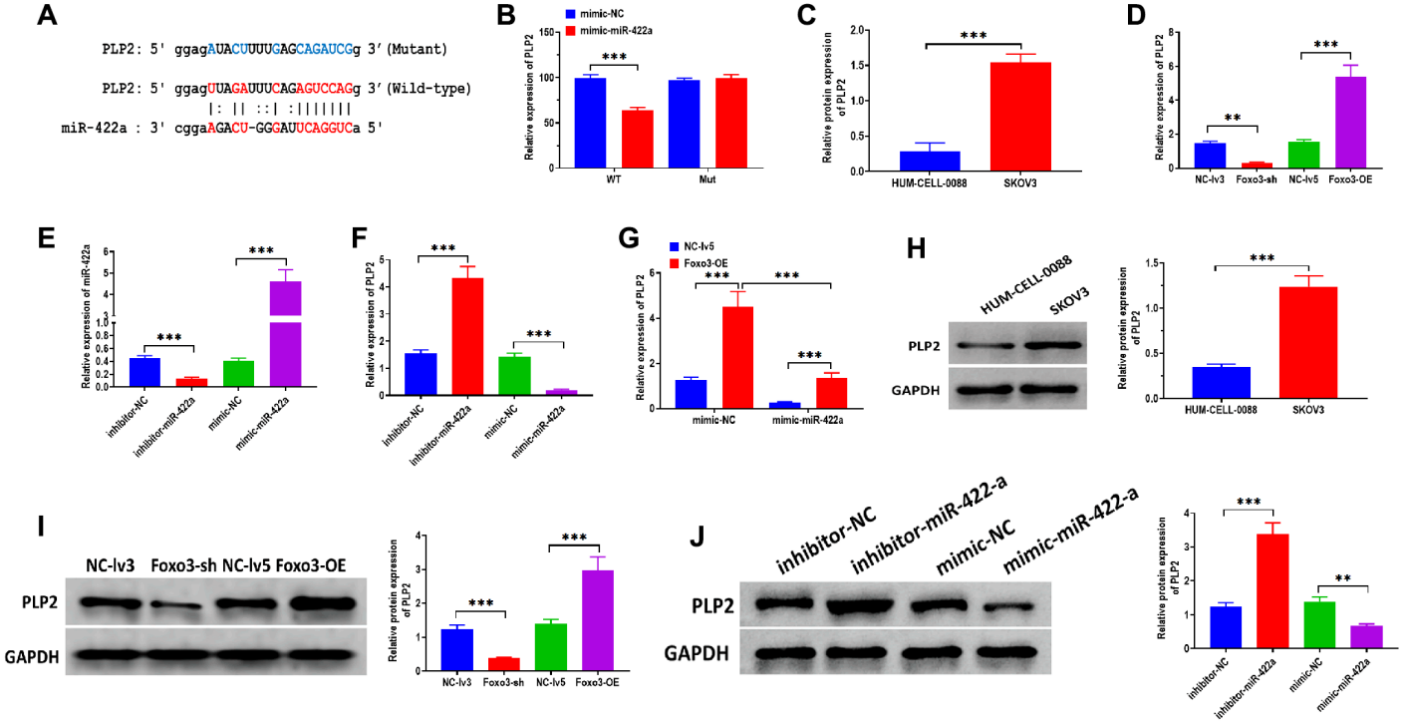
**miR-422a directly targets PLP2.** (**A**) The putative binding sequence of miR-422a of 3’UTR of PLP2. The prediction was obtained from StarBase 2.0. (**B**) Luciferase reporter assay in HEK293T cells co-transfected with miR-422a mimics or mimics a negative control plus reporter vector containing wild-type or mutant miR-422a binding sequence 3′-UTR of PLP2. (**C**) The mRNA expression of PLP2 in SKOV3 and control cells. (**D**) The mRNA expression of PLP2 in SKOV3 cells with Foxo3 knockdown or overexpressing. (**E**) Transfection efficiency in miR-422a-knockdown or miR-422a-overexpressing SKOV3 cells. (**F**) The mRNA expression of PLP2 in SKOV3 cells with miR-422a knockdown or overexpressing. (**G**) The mRNA expression of PLP2 in SKOV3 cells co-transfected with Foxo3-overexpressing and miR-422a-overexpressing vector. (**H**) The protein expression of PLP2 in SKOV3 and control cells. (**I**) The protein expression of PLP2 in SKOV3 cells with Foxo3 knockdown or overexpressing. (**J**) The protein expression of PLP2 in SKOV3 cells with miR-422a knockdown or overexpressing. Data were expressed as means ± standard deviation. For each treatment, three replicates were used. ^**^p < 0.01, ^***^p < 0.001.

Growing studies demonstrate that miRNAs exert essential biological roles mainly through targeting 3′UTR of mRNAs, thereby silencing genes [24, 25]. PLP2 has been reported to be a target gene of miR-422a and is associated with regulating breast cancer [21]. By bioinformatics analysis, there was a binding site of miR-422a in 3′UTR of PLP2 (Figure 4A). The luciferase reporter assay showed that the relative luciferase activity decreased in HEK293T cells co-transfected with miR-422a mimics and vectors containing wild-type binding sites miR-422a (Figure 4B). No luciferase activity change was observed in cells co-transfected with miR-422a mimics and vectors containing the mutant binding site. In addition, we found that PLP2 mRNA and protein expression was higher in SKOV3 cells compared with the control cells (Figure 4C and 4H). Both mRNA and protein expressions of PLP2 were increased in SKOV3 cells with Foxo3 overexpression and decreased in cells with Foxo3 knockdown (Figure 4D and 4I). Also, we applied miR-422a inhibitor and mimic to knockdown and overexpress miR-422a, respectively (Figure 4E). As we expected, the mRNA and protein levels of PLP2 were upregulated in cells treated with miR-422a inhibitor, whereas downregulated in cells treated with miR-422a mimic (Figure 4F and 4J). Furthermore, the expression of PLP2 was enhanced in cells co-transfected with Foxo3-OE vectors and miR-422a mimic negative control while suppressed in cells co-transfected with Foxo3-OE vector negative control and miR-422a mimic (Figure 4G). Collectively, these results suggest that PLP2 is a target gene of miR-422a and that Foxo3 may positively regulate PLP2 through targeting miR-422a.

### Foxo3 is transported through exosome-mediated intercellular communication

**Figure 5:**
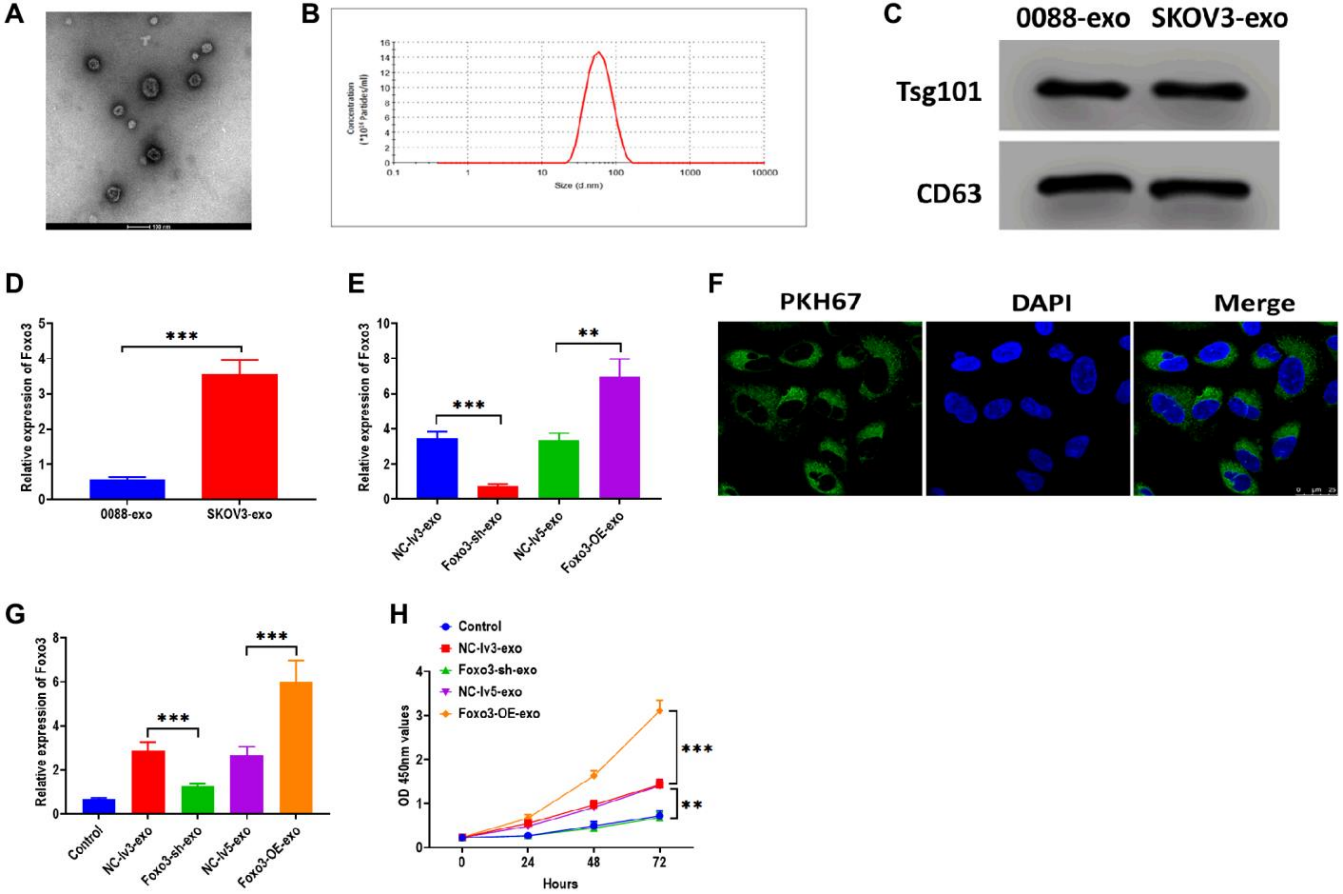
**Foxo3 is transported through exosome-mediated intercellular communication.** (**A**) The morphology of exosomes, as determined by TEM. Scale bar = 100 μm. (**B**) The size distribution of exosomes, as determined by NTA. (**C**) The protein expressions of exosomal markers Tsg101 and CD63 in exosomes isolated from SKOV3 and control cells. (**D**) The expression of Foxo3 in exosomes derived from the control or SKOV3 cells. (**E**) The expression of Foxo3 in exosomes with Foxo3 knockdown or overexpressing. (**F**) Visualization of exosomes taken up by SKOV3 cells, as observed by fluorescence microscope. Scale bar = 25 μm. (**G**) The expression of Foxo3 in SKOV3 cells co-cultured with exosomes with Foxo3 knockdown or overexpressing. (**H**) Cell proliferation of SKOV3 cells co-cultured with exosomes with Foxo3 knockdown or overexpressing, as determined by CCK-8 assay. Data were expressed as means ± standard deviation. For each treatment, three replicates were used. ^**^p < 0.01, ^***^p < 0.001.

**Figure 6:**
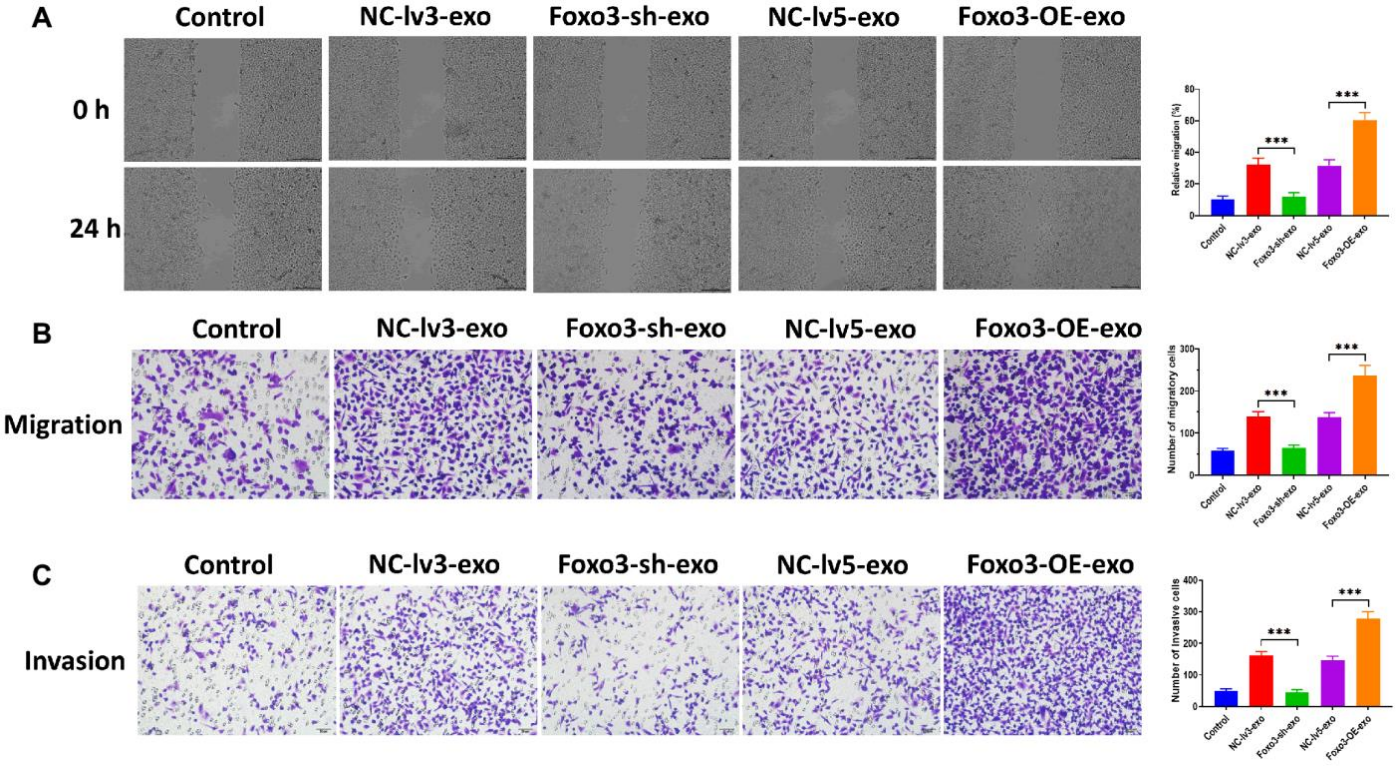
**Exosomal Foxo3 promotes OC cell migration and invasion.** (**A**) Migration ability of SKOV3 cells co-cultured with exosomes with Foxo3 knockdown or overexpressing, as determined by wound healing assay. Scale bar = 200 μm. (**B**) Migration ability of SKOV3 cells co-cultured with exosomes with Foxo3 knockdown or overexpressing, as determined by Transwell migration assay. Scale bar = 50 μm. (**C**) Invasion ability of SKOV3 cells co-cultured with exosomes with Foxo3 knockdown or overexpressing, as determined by Transwell invasion assay. Scale bar = 50 μm. Data were expressed as means ± standard deviation. For each treatment, three replicates were used. ^*^p < 0.05, ^***^p < 0.001.

Exosomes are essential for intercellular communication in cancer cells [26, 27]. In this study, we sought to investigate whether Foxo3 could be shuttled by exosomes, thereby regulating recipient cells’ biological processes. We isolated exosomes from the culture median of HUM-CELL-0088 and SKOV3 cells. The TEM and NTA assay revealed that exosomes showed a typical round-shape membrane structure, with 45-135 nm in diameter (Figure 5A and 5B). The western blotting assay showed that exosomes positively expressed exosomal markers Tsg101 and CD63 (Figure 5C). Next, we found that the level of Foxo3 was significantly higher in exosomes derived from SKOV3 cells (SKOV3-exo) compared with exosomes isolated from HUM-CELL-0088 cells (0088-exo) (Figure 5D). Also, the level of exosomal Foxo3 was increased in exosomes derived from SKOV3 cells with Foxo3 overexpression (Foxo3-OE-exo) while decreased in exosomes derived from cells with Foxo3 knockdown (Foxo3-sh-exo) (Figure 5E). Coculturing SKOV3 cells with exosomes, fluorescence staining assay revealed that PKH67-labeled exosomes were taken up by SKOV3 cells (Figure 5F). For SKOV3 cells co-cultured with exosomes as indicated, the level of Foxo3 was increased in cells treated with Foxo3-OE-exo while decreased in cells co-cultured with Foxo3-sh-exo (Figure 5G). Functionally, SKOV3 cells treated with Foxo3-OE-exo displayed enhanced cell proliferation, migratory, and invasive ability, and the opposite results were observed in cells treated with Foxo3-sh-exo (Figure 5H, Figure 6A–6C). Collectively, Foxo3 can be transported by exosomes to SKOV3 cells and regulates the progression of SKOV3 cells.

## DISCUSSION

OC is a lethal malignancy disease ranking the seventh most frequent cancer diagnosis worldwide and the eighth leading cause of cancer-associated death [28]. Thus, it is an urgent need to understand the progression of OC and explore the effective strategy for OC treatments. Given the vital role of Foxo3 in other cancer types, we focused on the function of Foxo3 in OC progression and the underlying mechanism. Our study demonstrates that Foxo3 was upregulated in OC cells and could promote OC cell proliferation, migration, and invasion through miR-442a/PLP2 signaling axis. Our results also indicate that Foxo3 could be transported by exosomes and participated in intercellular communication between OC cells.

Foxo3 and linear Foxo3 (Foxo3 mRNA) are both encoded from the Foxo3 gene, and their expressions are independent of each other [29, 30]. Currently, a growing body of evidence demonstrates that Foxo3 aberrantly expresses in several cancers, including breast cancer [31], lung cancer [12], and esophageal squamous cell cancer progression [10]. In this study, we also uncovered the critical role of Foxo3 in OC cells. In particular, the upregulation of Foxo3 is associated with enhanced OC cell proliferation, migration, and invasion, indicating the potential role of Foxo3 in OC progression. Intriguingly, our observation that Foxo3 was upregulated in OC is inconsistent with previous reports that Foxo3 acts as an anti-tumor factor in cancers [11, 12, 31]. Zhang and colleagues report that Foxo3 is downregulated in non-small cell lung cancer (NSCLC) tissues and cell lines, and the overexpression of Foxo3 promotes NSCLC cell apoptosis while inhibits cell proliferation, migration, and invasion through sponging miR-155 [12]. In bladder cancer, Foxo3 is decreased *in vitro* and *in vivo* and promotes apoptosis through targeting miR-191 [11]. On the other hand, Foxo3 is elevated in gastric cancer (GC) cells and promotes GC malignancy *in vitro* and *in vivo* by enhancing USP44 expression via targeting miR-143-3p [32]. A similar oncogenic role of Foxo3 has also been uncovered in prostate cancer [33] and glioblastoma [34]. Furthermore, Du et al. reported that the unregulated expression of Foxo3 is observed in over-confluent cancer cell culture, contributing to tumor development [30]. Collectively, the function and expression pattern of Foxo3 display a cell-type-dependent manner, indicating the multifunctional property of Foxo3.

As a novel group of ncRNAs, the detailed function of circRNAs has not been comprehensively investigated. So far, a well-known functional mechanism of cricRNAs is to regulate gene expression by interacting with miRNAs or RNA-binding proteins [35]. To further explore the mechanism underlying the role of Foxo3 in OC, we attempted to screen the potential targeting miRNAs of Foxo3. As the results suggested, Foxo3 could directly target miR-422a and negatively regulate its expression. Many previous studies have reported that miR-422a functions as an anti-tumor regulator in several cancers. For example, miR-422 was downregulated in colorectal cancer cells to inhibit cell proliferation [36]. The downregulation of miR-422a is associated with cell migration and cellular metabolism in gastric cancer [23]. Furthermore, the upregulation of miR-422a suppressed microsphere formation and tumor development of breast cancer stem cells [21]. Taken together, miR-422a may be a promising biomarker for developing treatments for cancers.

Growing evidence suggests that miRNAs play essential biological roles mainly through targeting 3′UTR of mRNAs, thereby silencing genes [24, 25]. PLP2 has been reported to be a target gene of miR-422a and is associated with regulating breast cancer [21]. In the present study, we also uncovered that miR-442a could directly bind to PLP2 and negatively regulate PLP2 expression. As a 4-transmembrane protein. PLP2 has been demonstrated to be elevated in multiple cancers, such as hepatocellular carcinoma [37], breast cancer [38], and osteosarcoma [39]. In addition, PLP2 also participates in several essential cellular processes, including invasion, adhesion, and proliferation, through PI3K/AKT signaling pathway [40]. However, the detailed regulatory role of PLP2 in these processes remains unclear. Thus, PLP2 may provide a new window to understand the cellular mechanism of cancer progression.

Exosomes can be secreted by almost all cell types and are widely present in various body fluids, such as saliva and blood [13]. There is plenty of evidence reporting the essential role of exosomes in the initiation and progression of cancer [41]. In particular, exosomes play a remarkable role in intercellular communication by shuttling bioactive cargos, including circRNAs, further regulating recipient cells’ biological processes and cancer development [42, 43]. To date, rare studies have reported the function of exosomal circRNAs in OC progression. In this study, we found that Foxo3 was highly expressed in exosomes derived from OC cells. Also, exosomes could be taken up by OC cells, and the level of Foxo3 was notably increased in recipient cells. Furthermore, exosomes with Foxo3 overexpression and knockdown could promote and inhibit OC progression, respectively. These results together suggest that the pro-tumor effect of Foxo3 is mediated by exosome-based cell-to-cell communication.

## CONCLUSIONS

In conclusion, the results demonstrate that Foxo3 promotes OC progression by miR-422a/PLP2 axis and that the effect of Foxo3 was also mediated by exosome-based intercellular communication. This study provides a novel understanding of the function of circRNAs in OC progression. Foxo3 may serve as a promising biomarker for further preclinical research.

## MATERIALS AND METHODS

### Cell culture

The human ovarian cancer cell lines SKOV-3, TOV-21G, OV-90, OVCAR-3, and human embryonic kidney cell line HEK293T were obtained from the American Type Culture Collection (USA). Cells were cultured in Dulbecco’s modified Eagle’s medium (DMEM)/F12 medium (Gibco, USA) supplemented with 10% fetal bovine serum (FBS) (Gibco, USA), 100 U/ml streptomycin, and 100 U/ml penicillin. HEK293T at 37°C in a 5% CO_2_ under saturated humidity.

### Quantitative reverse transcription-polymerase chain reaction (qRT-PCR)

Total RNA was isolated from cells using TRIzol reagent (Invitrogen, USA) or exosomes using Exosomal RNA and Protein Extraction Kit (101Bio, USA) according to the manufacturers’ instructions. The concentration and quality of total RNA were determined using NanoDrop™ 1000 Spectrophotometer (NanoDrop Technologies, USA). First-strand cDNAs were synthesized using the Goscript™ Reverse Transcription System (Promega, USA) according to the manufacturers’ instructions. qRT-PCR was performed using the qPCR SYBR Green Mix (Bio-Rad, USA) on the Applied Biosystems 7500 Real-Time PCR System (Applied Biosystems, USA) based on the recommended reaction conditions. GAPDH and U6 were used as internal reference genes. The primers used in this study were as follows: GAPDH: (F) 5′-AGAAGGCTGGGGCTCATTTG-3′ and (R) 5′-AGGGGCCATCCACAGTCTTC-3′; U6: (F) 5′-CTCGCTTCGGCAGCACA-3′ and (R) 5′-AACGCTTCACGAATTTGCGT-3′; Foxo3: (F) 5′-GGCCTCATCTCAAAGCTGG-3′ and (R) 5′-CTTGCCCGTGCCTTCATT-3′; miR-422a: (F) 5′-GCACTGGACTTAGGGTCA-3′ and (R) 5′-TGGTGTCGTGGAGTCG-3′; proteolipid protein 2 (PLP2): (F) 5′-CTCATAGCGGCAATCCTCTAC-3′ and (R) 5′-AAGGTGACATAGGCATCATAGC-3′. PCR reactions were carried out in triplicate. Data were analyzed using the 2^−ΔΔCt^ method [44].

### Cell transfection

Lentivirus-3 Foxo3-shRNA (Foxo3-sh), lentivirus-5 Foxo3 (Foxo3-OE), and respective negative vectors were synthesized by Shanghai GenePharma Co., Ltd. (Suzhou, China). miR-422a mimics, inhibitors, and respective negative controls were purchased from RiboBio (Guangzhou, China). The sequence information was as follows: Foxo3-sh: 5′-CTTGGACCTGGACATGTTCAATGG-3′; Foxo3-OE: 5′-TTTCGGATCCTTC AGCCTGGCACC-3′; miR-422a mimic: 5′-ACUGGACUUAGGGUCAGAAGGC-3′; miR-422a inhibitor: 5′-GCCUUCUGACCCUAAGUCCAGU-3′. SKOV-3 cells (2 × 10^5^) were seeded in a 6-well plate for 12 h for attachment. Then, cells were transfected with respective vectors using Lipofectamine™ 2000 (Thermo Fisher Scientific, USA) according to the manufacturer’s instruction. The transfection efficiency was determined by qRT-PCR assay.

### CCK-8 assay

Cells (2 × 10^5^) were inoculated in 96-well plates (Corning, USA) and cultured for 24 h, 48 h, and 72 h, respectively. At each time point, 10 μL from CCK-8 kit reagent (Dojindo, Japan) was added to each well and incubated for 1 h according to the manufacturer’s instruction. The cell proliferation was measured using the microplate reader (Bio-Rad, USA) at 450 nm absorbance.

### Wound healing assay

Cells (2 × 10^5^) in the logarithmic growth phase were seeded in a 6-well plate (Corning, USA). When the cells reached over 90% confluence, a scratch was made using a 200 μL pipette tip. Then the cells were washed three times with PBS to remove the floating cells. Cells or exosome-treated cells were cultured with DMEM/F12 medium (serum-free) (Gibco, USA) at 37°C and 5% CO2 for 24 h. Images were taken at 0 and 24 h under a TS100-F inverted microscope (Nikon, Japan).

### Transwell migration and invasion assay

Cells (2 × 10^5^) were suspended in DMEM/F12 medium supplemented with 10% FBS and then were seeded in a 24-well plate (700 μL/well). Transwell chambers with 8 μm pore size (BD Biosciences, USA) were placed on the wells. Cells were placed into the upper compartment of the Transwell chamber. After 24 h, cells passed through the membrane were fixed with methanol and stained with 0.1% crystal violet solution for 20 mins. Images were taken under a TS100-F inverted microscope (Nikon, Japan). The migratory and invasive cells were counted in five random fields per well.

### Western blotting

Total protein was isolated from cells using RIPA Lysis Buffer (Beyotime, China) or exosomes using Exosomal RNA and Protein Extraction Kit (101Bio, USA) according to the manufacturer’s instruction. Total protein was quantified using the BCA Protein Assay Kit (Thermo Fisher Scientific, USA). The western blotting assay was performed as previously described [45]. Antibodies used in this study were as follows: CD63 (1:1000), Tsg101 (1:1000), GAPDH (1:2000), and PLP2 (1:1000) (Santa Cruz Biotechnology, China). The protein identity was visualized by the ECL chemiluminescence reagent (Millipore, USA) and analyzed using an Odyssey Imaging System (LI-COR, USA).

### Dual-luciferase reporter assay

Luciferase reporter vectors (Foxo3/PLP2-WT, Foxo3/PLP2-Mut) were constructed by Promega (USA). Foxo3/PLP2-WT or Foxo3/PLP2-Mut vectors, miR-422a mimics, or negative control miRNAs were co-transfected into HEK293T cells using Lipofectamine™ 2000 (Thermo Fisher Scientific, USA) according to the manufacturer’s instruction. The relative luciferase activity was determined using the Dual-luciferase Reporter Assay System (Promega, USA) according to the manufacturer’s instruction.

### Subcellular fractionation assay

Nuclear and cytoplasmic RNA isolation in SKOV-3 cells was carried out as previously described [46]. The expression of Foxo3 in each cellular fraction was determined by qRT-PCR.

### Exosome isolation and identification

Exosomes in FBS were removed by ultracentrifugation prior to experiments. Exosome was isolated from cell culture supernatant using ultracentrifugation method as previously described [47, 48]. Transmission electron microscopy (TEM) was used to visualize exosomes’ morphology, as previously described [49]. The concentration and size distribution of exosomes were determined by Nanoparticle tracking analysis (NTA) via using NanoSight NS500 (Malvern Instruments, UK) according to the manufacturer’s instruction [50, 51].

### Fluorescence *in situ* hybridization (FISH)

1 μg/ml exosomes were suspended in 100 μl PBS solution with 1 ml PKH67 dye (Sigma, USA). After incubation for 4 mins at room temperature, the staining process was terminated by the addition of bovine serum albumin (BSA) (Sigma-Aldrich, USA). Labeled exosomes were extracted using Exoquick exosome precipitation (System Biosciences, USA) according to the manufacturer’s instructions. Exosomes were co-cultured with SKOV-3 cells labeled with DAPI (Thermo Fisher Scientific, USA) for 3h at 37°C. Images were taken using a laser confocal microscope (Leica, Germany).

### Statistical analysis

Data were presented as means ± standard deviation and analyzed using SPSS 22.0 software (SPSS Inc, USA). All experiments were conducted with at least three independent biological replicates. Differences between groups were analyzed with Student’s *t*-test or one-way analysis of variance (ANOVA). *p* < 0.05 indicated a statistically significant difference.
